# Selective Redox Tuning Enables Potent Intracellular Reduction of Nicotinamide Cytosine Dinucleotide

**DOI:** 10.3390/biom16091255

**Published:** 2026-08-29

**Authors:** Xiaojia Guo, Yanzhe Huang, Yinghan Hu, Lingyun Zhang, Zongbao K. Zhao

**Affiliations:** 1 MOE Key Laboratory of Bio-Intelligent Manufacturing, School of Bioengineering, Dalian University of Technology, Dalian 116024, China; guoxiaojia@dlut.edu.cn; 2 Dalian Institute of Chemical Physics, Chinese Academy of Sciences, Dalian 116023, China; huangyz@dicp.ac.cn (Y.H.); huyinghan@dicp.ac.cn (Y.H.); zhanglingyun21@dicp.ac.cn (L.Z.)

**Keywords:** nicotinamide cytosine dinucleotide, selective redox regulation, redox cofactor, energy transfer, phosphite dehydrogenase

## Abstract

The ubiquitous nicotinamide adenine dinucleotide (NAD) engages in diverse biological processes, leading to non-selective energy transfer toward target synthetic reactions. To achieve selective energy transfer in complex biological systems, we previously constructed artificial systems mediated by the non-natural cofactor nicotinamide cytosine dinucleotide (NCD), which can be specifically recognized by engineered enzymes with minimal cross-talk with natural cofactors. For enhanced energy transfer and higher product yields, efficient conversion of NCD to NCDH is required to deliver reducing power in NCD-mediated biosynthetic pathways. Here, we established a comprehensive strategy for selective reduction in intracellular NCD. First, coupled enzymatic colorimetric assays with high specificity were validated for quantifying NAD, NADP, and NCD. With phosphite as the energy source, we selectively elevated the intracellular NCDH/NCD ratio with minimal perturbation to NADH/NAD and NADPH/NADP ratios in *E. coli*. To overcome the limitation of phosphite transmembrane transport, cell-free systems were constructed to confirm that phosphite could drive near-complete NCD reduction. Finally, cells were treated with polymyxin B, which promoted phosphite uptake and thereby enabled maximal reduction in intracellular NCD. An NCDH/NCD ratio of 47 was achieved, demonstrating that 98% of the intracellular NCD pool existed in the reduced form. This work demonstrates that NCD can function as an independent redox cofactor for selective regulation, providing viable strategies for artificial cofactor-driven biosynthesis.

## 1. Introduction

Exploration of redox cofactors stands as a prominent research frontier in biocatalysis and synthetic biology. A large number of oxidoreductases employ nicotinamide adenine dinucleotide (NAD) or nicotinamide adenine dinucleotide phosphate (NADP) as the cofactor to mediate electron transfer in diverse reactions, such as NAD(P)-dependent enzymes for the biosynthesis of chiral amines, chiral alcohols and amino acids [1,2,3,4].

The NAD(P) pool is critical to numerous reactions in intracellular biosynthesis. Maintaining a continuous and stable supply of NAD(P) or its reduced form NAD(P)H is a fundamental challenge for efficient bioproduction [5,6,7,8]. Regulating and balancing NAD(P) pools have emerged as a universal strategy to enhance target metabolic flux and improve production [9,10]. For instance, cofactor modulation serves as a driving force for the biosynthesis of fatty alcohols, flavonoids, and related metabolites. Enhancing NAD(P)H supply by overexpressing NAD(P)-dependent isocitrate dehydrogenase and introducing a malate cycle improved fatty alcohol production in *Ogataea polymorpha* [11]. By regulating cofactor supply and improving the pool of NADH and NADPH, enhanced titers of *p*-Coumaric acid and nylon 12 monomer were achieved in *E. coli* [12,13].

**Scheme 1:**
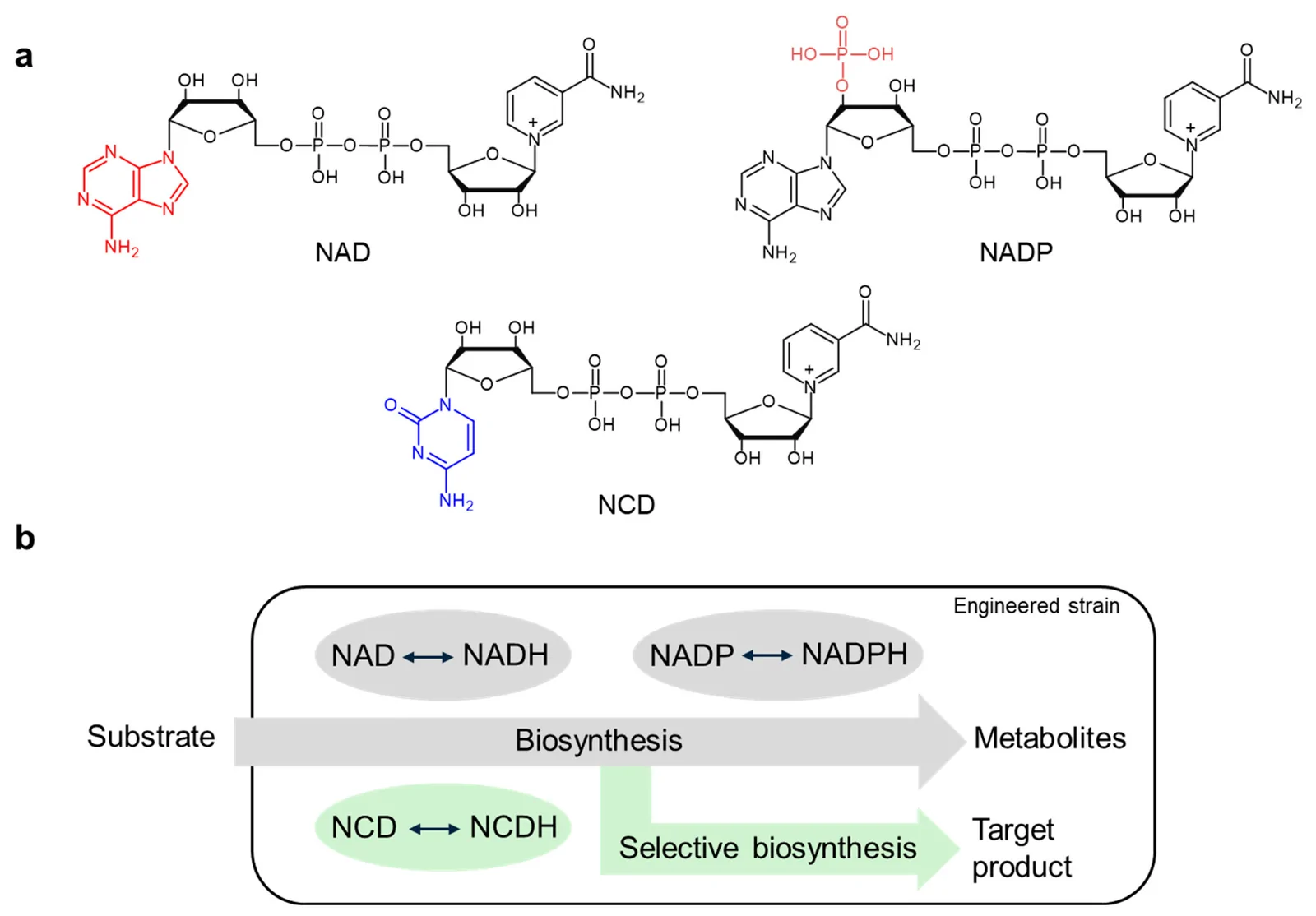
NAD, NADP, and NCD participate in biosynthesis. (**a**) Structures of NAD, NADP, and NCD. Structural differences between NAD, NADP, and NCD are highlighted in distinct colors. (**b**) NCD(H) drives the selective synthesis of the target product.

However, NAD(P) is involved in over 1500 enzymatic reactions in microbial cells [14]. The ubiquitous participation of NAD(P) in metabolic networks makes it difficult to selectively transfer reducing power to the target reaction [15]. NAD analogs offer promising alternatives for the dedicated regulation of target reactions in vivo, as they are specifically recognized by the engineered enzymes [16]. Recently, nicotinamide mononucleotide (NMN), a precursor in NAD biosynthesis, was utilized as a non-canonical cofactor in the biosynthesis of 2,3-butanediol when coupled with an engineered butanediol dehydrogenase [17]. In our previous work, we employed a non-natural cofactor, nicotinamide cytosine dinucleotide (NCD), as an energy carrier (Scheme 1a), and acquired a series of enzymes that preferred NCD [18,19]. Through directed evolution, we created an NCD synthetase and constructed an NCD self-sufficient *E. coli* chassis, and NCD-mediated systems were further developed with both NCD and NCDH regeneration modules for selective energy regulation [20]. In NCD self-sufficient strains, NCD functions as the third nicotinamide cofactor, which supports the selective synthesis of target products (Scheme 1b). Similar to NAD and NADP, regulation of the NCD pool is important to NCD-mediated biosynthesis, and the intracellular NCDH/NCD ratio serves as an important indicator reflecting whether the NCD pool exists in an oxidized or reduced state. In synthetic pathways involving reductive reactions, the NCDH/NCD ratio provides an important reference for tuning NCDH availability. However, the regulation of the NCD pool and the extent to which the NCDH/NCD ratio can be modulated have not been fully elucidated.

Polymyxins are a group of basic polypeptide antibiotics, consisting of a polycationic peptide ring and a tripeptide side chain containing a fatty acid tail. Polymyxin B interacts with lipopolysaccharides on the outer membrane of Gram-negative bacteria. The polycationic peptide ring of polymyxin B displaces calcium and magnesium ions from lipopolysaccharides, while the fatty acid chain inserts into the outer membrane structure, altering membrane permeability and creating transient pores [21,22]. In this study, we achieved selective regulation of the NCDH/NCD ratio with the assistance of polymyxin B. We first established and validated a set of coupled enzymatic colorimetric assays for specific quantification of the NCD, NAD, and NADP. Then, phosphite was used as an energy source to selectively tune the intracellular NCDH/NCD ratio by using the NCD-dependent Pdh* [19]. Complete reduction in NCD was then conducted in the cell-free systems, bypassing the intracellular uptake limitation of phosphite. Further, polymyxin B-induced cell permeabilization was applied to enhance phosphite uptake, achieving robust reduction in intracellular NCD, with the NCDH/NCD ratio reaching 47. This work develops a feasible strategy for selective redox control of the non-natural cofactor NCD, and lays a solid foundation for constructing NCD-dependent biosynthetic pathways.

## 2. Materials and Methods

### 2.1. Strains and Media

Strains used in this study were listed in Table S1. The plasmid pk-2B3-1G6 carrying phosphite dehydrogenase mutant I151R/P176R/M207A [19] was utilized to construct engineered strains. The plasmid pUC-WXY63, which harbors the NCD synthesis module, was used for NCD biosynthesis, while pUC-P15A served as the negative control for NCD synthesis [20]. Plasmid pCA24N-FbaA from the ASKA library was used for the expression and purification of FbaA [23]. LB medium and TB medium were used for strain culture and protein expression. MOPS medium was used for cofactor regulation. MOPS medium contained 40 mM MOPS, 4 mM tricine, 0.01 mM FeSO_4_, 9.5 mM NH_4_Cl, 0.276 mM K_2_SO_4_, 5 × 10^−4^ mM CaCl_2_, 0.528 mM MgCl_2_ and 50 mM NaCl, with the pH adjusted to 7. Glucose was supplemented at a final concentration of 4 g/L, and 2 mM sodium phosphite was added when required.

### 2.2. Cofactor Determination

For NCD determination, the system contained 50 mM HEPES (pH 7.5), 10 mM sodium formate, 0.4 mM thiazolyl blue (MTT), 1 mM phenazine ethosulfate (PES), 0.27 mg/mL NCD-dependent formate dehydrogenase mutant (V198I/C256I/P260S/E261P/S381N/S383F) [18] and 10 μL NCD or mixed cofactors. The assays for NAD determination contained 100 mM Bicine (pH 8.0), 10 μL ethanol, 0.4 mM MTT, 1 mM PES, 4 mM EDTA, 5 U/mL alcohol dehydrogenase (ADH) (Sigma-Aldrich, St. Louis, MO, USA), and 10 μL NAD or mixed cofactors. For NADP, the system contained 50 mM HEPES (pH 7.5), 10 mM fructose 1,6-bisphosphate, 0.4 mM MTT, 1 mM PES, 0.07 mg/mL fructose-bisphosphate aldolase class II from *E. coli* (FbaA) (Gene ID: 947415) [23], 0.05 mg/mL glyceraldehyde-3-phosphate dehydrogenase from *Streptococcus mutans* (*Sm*GAPN) (GenBank: AAN58410.1), and 10 μL NADP or mixed cofactors. Absorbance was monitored with a microplate reader (BioTek Instruments, Winooski, VT, USA) at 570 nm continuously. NCD was synthesized in this study as previously reported [24]. Calibration curves for NCD and NAD were constructed with serial concentrations (μM): 10, 20, 40, 80, 160, and 320. The standard curve of NADP was established using concentrations (μM) of 0.3125, 0.625, 1.25, 2.5, 5, 10, 20, and 40. All measurements were performed at 30 °C. Intracellular cofactor concentration was calculated according to the following equation:

$$\mathrm{Intracellular}\;\mathrm{cofactor}\;\mathrm{concentration}\;=\frac{c\;\times \mathrm{Vs}}{N\times Vc}$$

where c is the concentration of the cofactor in the detected sample; Vs, sample volume, 0.2 mL; N, total number of cells; Vc, sample volume of *E. coli*, 10^−12^ mL [25]. An OD_600_ of 1 corresponds to a cell density of 10^9^ cells/mL, as determined by serial dilution of the culture and colony counting on agar plates.

### 2.3. Cofactor Extraction

A total of 1 × 10^10^ cells were collected in triplicate by adjusting the culture volume based on OD_600_ (OD_600_ × volume = 10), and washed with 0.5 mL of 40 mM MOPS (pH 7.2). For oxidized cofactor extraction, cell pellets were first resuspended in 100 μL 0.2 M HCl, and then incubated at 55 °C for 10 min, followed by supplementation with 100 μL of 0.1 M NaOH. The mixture was centrifuged at 12,000 *g* and 4 °C for 5 min, and the supernatant was collected for quantification. For the extraction of reduced cofactors, cell pellets were resuspended in 100 μL 0.2 M NaOH, and then incubated at 55 °C for 10 min, followed by supplementary of 100 μL of 0.1 M HCl. The mixture was centrifuged at 12,000 *g* and 4 °C for 15 min, and the supernatant was used for quantification. The stability of NCD and NCDH during the extraction procedure was verified by processing standard cofactors through the same steps and comparing them with untreated controls using the colorimetric assays.

### 2.4. Determination of Crude Enzyme Activity

A total of 1 × 10^10^ cells were collected (based on the correlation of OD_600_ × volume = 10) and lysed in 0.25 mL cell lysis solution containing 10 mM Tris (pH 8.0), 1 mM MgCl_2_, 1 mg/mL lysozyme, and 0.1 mg/mL DNase I at 37 °C and 200 rpm for 2 h. Samples were then centrifuged, and the supernatant was used as the crude enzyme. Activity of the crude enzyme was determined in a volume of 100 μL with 50 mM HEPES (pH 7.5), 100 μM cofactor, 5 mM phosphite, 0.4 mM MTT, 1 mM PES, and 10 μL crude enzyme. Absorbance was measured at 570 nm continuously.

### 2.5. Cofactor Regulation

Cells were cultured in 7.5 mL LB medium supplemented with kanamycin (50 μg/mL) and carbenicillin (50 μg/mL) at 37 °C for 12 h. Subsequently, the culture was inoculated at a dilution ratio of 1:100 into 50 mL TB medium containing kanamycin (50 μg/mL), carbenicillin (50 μg/mL), IPTG (1 mM), and L-arabinose (1 mM), followed by incubation at 30 °C with shaking at 200 rpm for 22 h. In the assays to examine whether phosphite could be utilized by engineered strains, no inducers were supplemented, and no antibiotics were added when cultivating the host strain BW14329 [26].

A total of 1 × 10^10^ cells (based on the correlation of OD_600_ × volume = 10) were collected and washed with 0.5 mL MOPS buffer (pH 7.2). For phosphite consumption assays, cell pellets were resuspended in 0.5 mL MOPS buffer (pH 7.2), and then incubated at 30 °C with shaking at 200 rpm. Samples were collected and centrifuged at 10,000 g and 4 °C for 2 min, and the supernatants were subjected to phosphite quantification. For the in vivo cofactor regulation experiments, cells were resuspended in 0.5 mL MOPS medium and incubated at 30 °C with shaking at 200 rpm. Samples were harvested for subsequent cofactor extraction. Cofactor regulation assays of permeabilized cells were performed in a 300 μL reaction mixture. The system contained 40 mM MOPS (pH 7.2) and 10 mM phosphite as needed. Different concentrations of polymyxin B were supplemented as required. Samples were incubated at 30 °C with shaking at 200 rpm. After centrifugation, cell pellets were collected for intracellular cofactor extraction, while the supernatants were used to determine extracellular cofactors released via membrane leakage.

For cell-free cofactor reduction assays, with the culture volume adjusted according to OD_600_ (OD_600_ × volume = 25), a total of 2.5 × 10^11^ cells were lysed in triplicate in cell lysis solution at 37 °C and 200 rpm for 3 h. A total reaction volume of 200 μL was used, containing 180 μL cell lysate, 5 mM phosphite and 50 μM cofactor as needed. Changes in absorbance at 340 nm were monitored continuously using a microplate reader at 30 °C.

### 2.6. Protein Purification

Proteins were expressed in *E. coli* BL21(DE3) and purified using a Ni-NTA kit (Invitrogen, Carlsbad, CA, USA). In brief, strains were cultivated at 37 °C for 12 h in LB medium containing antibiotics (50 μg/mL kanamycin for *Sm*GAPN and 34 μg/mL chloramphenicol for FbaA). The cultures were then transferred into TB medium supplemented with 0.1 mM IPTG and corresponding antibiotics, and incubated at 30 °C for 48 h. For *Sm*GAPN purification, 3 mM β-mercaptoethanol was added to purification buffers. For FbaA production, 0.3 mM ZnCl_2_ was supplemented in the TB medium. The formate dehydrogenase mutant was purified as reported [18]. Protein concentrations were quantified by the Bio-Rad protein assay kit using BSA as the standard, and SDS-PAGE analysis was used to assess protein purity (Figure S1).

### 2.7. Determination of Phosphite

Phosphite was determined by a Thermo Fisher ICS-5000+ ion chromatography system (Thermo Fisher Scientific, Sunnyvale, CA, USA) equipped with a suppressed conductivity detector. A Dionex IonPac AS11-HC analytical column (250 mm × 2 mm) (Thermo Fisher Scientific, Sunnyvale, CA, USA) and a Dionex IonPac AG11-HC guard column (50 mm × 4 mm) (Thermo Fisher Scientific, Sunnyvale, CA, USA) were used for analysis. Samples were analyzed by gradient elution. Specifically, from 0 to 20 min, 2 mM NaOH was used as the mobile phase. The concentration of NaOH was increased to 40 mM from 20 min to 30 min, and kept at 40 mM for 37 min. The system was equilibrated with 200 mM NaOH for 20 min before the next injection. Samples were determined at 30 °C with a flow rate of 1 mL/min. A 30 mM H_2_SO_4_ solution was used as the regenerant to ensure a stable baseline and optimal detector performance. Before injection, samples were centrifuged, and the supernatant was filtered through a 0.22 μm membrane. A calibration curve was constructed using serial dilutions of sodium phosphite standards, and the concentration of phosphite in the samples was determined according to the standard curve.

### 2.8. Statistical Analysis

Data are presented as mean ± standard deviation (SD). Statistical comparisons between two groups were performed using two-tailed Student’s *t*-tests. A *p*-value of <0.05 was considered statistically significant. Statistical analyses were performed using GraphPad Prism (version 11).

## 3. Results and Discussion

### 3.1. Validation of Enzymatic Assays for Cofactor Determination

**Figure 1:**
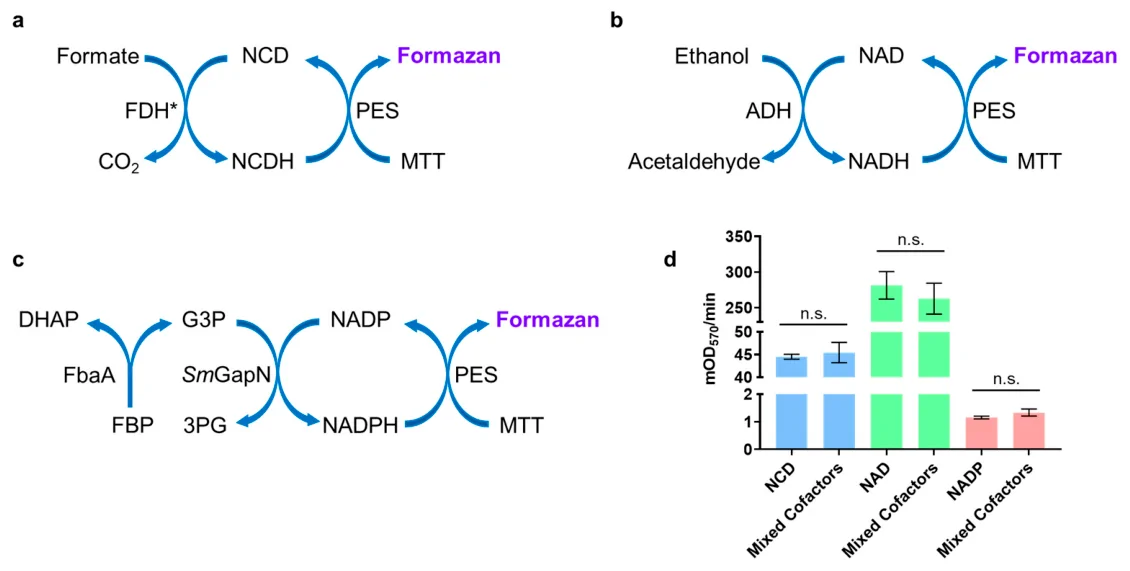
Principles and validation of cofactor quantification. (**a**–**c**) Principles of colorimetric assays. FDH*, formate dehydrogenase mutant V198I/C256I/P260S/E261P/S381N/S383F; ADH, alcohol dehydrogenase from *Saccharomyces cerevisiae*; FbaA, fructose-bisphosphate aldolase class II from *E. coli*; *Sm*GapN, glyceraldehyde-3-phosphate dehydrogenase from *Streptococcus mutans*. MTT, thiazolyl blue; PES, phenazine eDthosulfate; DHAP, dihydroxyacetone phosphate; FBP, D-fructose 1,6-bisphosphate; G3P, D-glyceraldehyde 3-phosphate; 3PG, 3-phospho-D-glycerate. (**d**) Validation of cofactor quantification methods. The total concentration of mixed cofactors was 320 μM with a molar ratio of NCD:NAD:NADP at 10:10:1. The concentration of each individual cofactor in the single-cofactor assay was identical to that of the corresponding cofactor in the mixed system. All measurements were conducted in triplicate. Data are shown as mean ± SD (n = 3). n.s. indicates no significant difference between the indicated groups (two-tailed Student’s *t*-test, *p* > 0.05).

To quantify the amount of NCD, NAD, and NADP, coupled enzymatic colorimetric assays were developed. In these assays, a specific enzyme was employed to reduce the oxidized cofactor, and the reduced cofactor was then recycled to the oxidized form with the transformation of MTT to formazan. Specifically, an NCD-dependent formate dehydrogenase mutant FDH* (V198I/C256I/P260S/E261P/S381N/S383F) [18] was used for NCD reduction. Residues near the adenine moiety of NAD in the binding pocket of the enzyme were subjected to mutagenesis. The resulting FDH* mutant possessed a more compact pocket that favors the smaller NCD. Meanwhile, new interactions were formed between the mutated residues and the cytosine moiety of NCD, conferring exclusive specificity for NCD over NAD and NADP. Therefore, even in complex systems where NAD and NADP are present, FDH* exclusively reduces NCD. ADH from *Saccharomyces cerevisiae* was used for NAD reduction. For NADP, fructose-bisphosphate aldolase class II (FbaA) from *E. coli* and NADP-dependent glyceraldehyde-3-phosphate dehydrogenase (*Sm*GapN) from *Streptococcus mutans* were coupled with D-fructose 1,6-bisphosphate (FBP) as the substrate [27]. The final product of these assays was formazan, which was then monitored at 570 nm (Figure 1a–c).

Although the aforementioned proteins FDH*, ADH, and *Sm*GapN exhibit preferential utilization of specific cofactors, the target cofactor to be quantified must remain free from interference by other cofactors in samples. For NAD and NCD quantification, the concentration gradient of the standard curve is set to range from 10 to 320 μM or 160 mM, according to the intracellular concentration of NAD and NCD at the micromolar level [20,28]. Given the intracellular NADP(H) concentration of approximately 0.12 mM [28], the standard curve was calibrated over a concentration range of 0.31 to 20 μM (Figure S2). Mixtures containing NAD, NCD, and NADP at a total concentration of 320 μM with a molar ratio of 10:10:1 were detected to evaluate cross-interference among these cofactors. It showed that absorbance changes over time at 570 nm between the single cofactor and the mixed cofactor revealed no significant differences (Figure 1d). Notably, the absorbance changes remained unchanged when NADP was increased to 40 μM when FDH* or ADH was used, indicating that these two enzymes were barely active toward NADP. Given that the assay is based on enzyme-coupled reactions, trace amounts of reduced cofactors added to the system are immediately transformed to the corresponding oxidized forms and subsequently participate in the reaction. Hence, this method allows simultaneous quantification of oxidized and reduced cofactors. The above results demonstrated that all developed systems possessed high specificity and were capable of quantifying the target cofactor in the presence of other cofactors. Compared with HPLC-based methods, our enzyme-coupled colorimetric approach offers higher throughput and does not require extensive sample pretreatment, making it particularly suitable for routine determination of cofactor levels in engineered strains.

### 3.2. Selective Regulation of Intracellular NCDH/NCD Ratio Driven by Phosphite as an Energy Source

Phosphite dehydrogenase (Pdh) oxidizes phosphite with the concurrent reduction of NAD to NADH. In our previous work, an NCD-dependent Pdh variant Pdh* (I151R/P176R/M207A) was obtained via directed evolution. The compact cofactor-binding pocket and the resulting interactions with NCD confer its exclusive specificity for NCD over NAD and NADP [19]. Hence, Pdh* was utilized to modulate the intracellular NCDH/NCD ratio. *E. coli* strain BW14329 has the *phn* operon and *phoA* gene knocked out [26], rendering it unable to utilize phosphite. Using BW14329 as the host, we next constructed engineered strains XJD2701 harboring Pdh* and the plasmid for NCD synthesis [20]. Strain XJD2801 harboring Pdh* was used as the negative control for NCD synthesis (Table S1).

**Figure 2:**
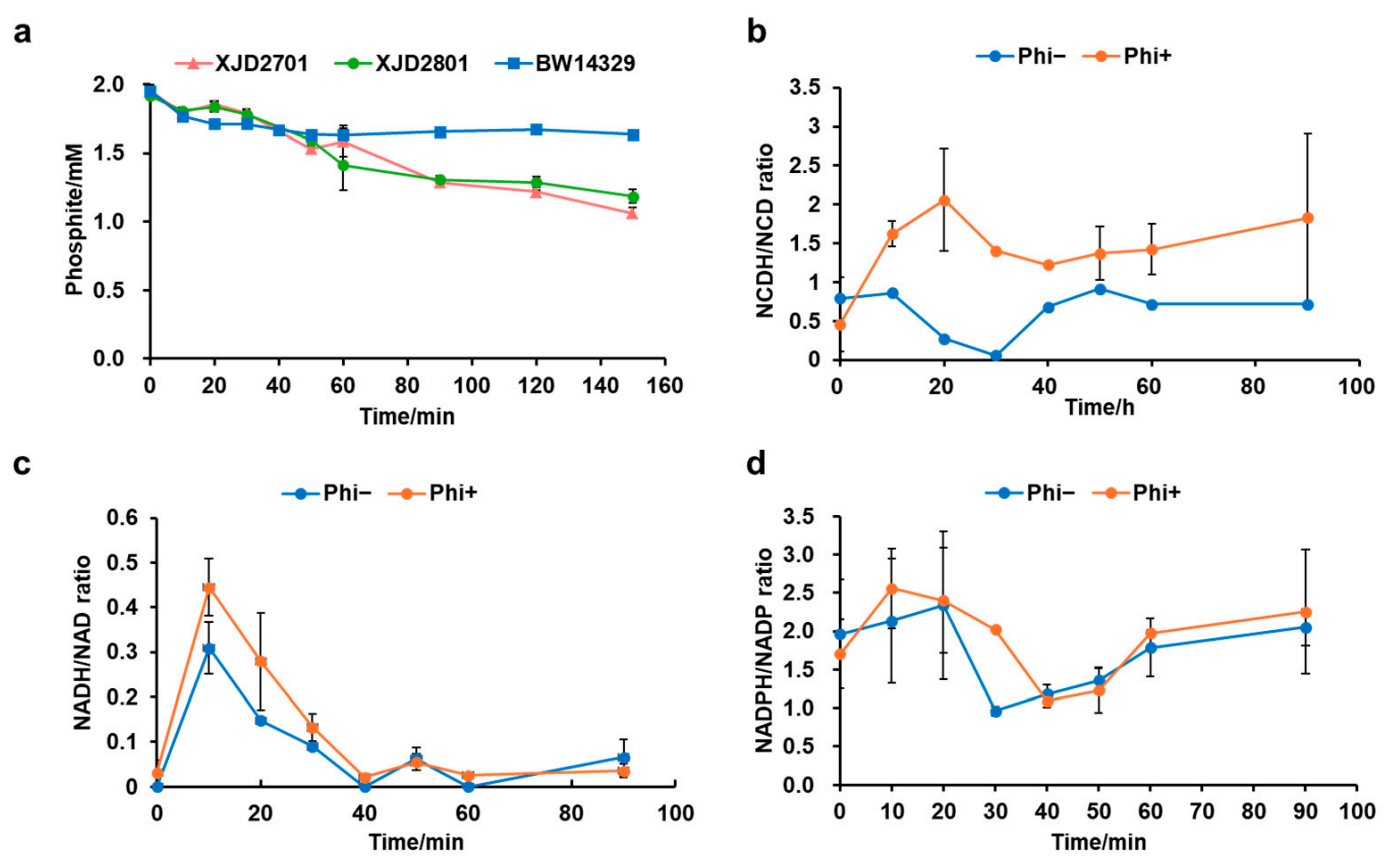
Phosphite consumption and cofactor ratios of the engineered strain. (**a**) Phosphite consumption over time of strain BW14329 (the host strain), XJD2701 (expressing Pdh* and NCD synthesis module), and XJD2801 (expressing Pdh*). (**b**) NCDH/NCD ratio of XJD2701. (**c**) NADH/NAD ratio of XJD2701. (**d**) NADPH/NADP ratio of XJD2701. Cells were suspended in 40 mM MOPS (pH 7.2) with or without phosphite supplementation. Phi−, without phosphite; Phi+, with phosphite. All measurements were conducted in triplicate.

Cells were suspended in MOPS buffer (pH 7.2) supplemented with 2 mM phosphite. In strain BW14329, the phosphite concentration in the system barely changed over time. In contrast, the phosphite level gradually decreased in the engineered strain expressing Pdh* (Figure 2a). This confirmed that BW14329 cannot utilize phosphite. During the detection in 150 min, 0.89 mM and 0.73 mM phosphite were consumed by strain XJD2701 and XJD2801, respectively, demonstrating enhanced phosphite utilization in the presence of NCD. The phosphite utilization in XJD2801 without NCD could be attributed to the retained activity of Pdh* towards the native cofactors NAD and NADP (Figure S3).

The ratios of reduced cofactor to oxidized cofactor in strain XJD2701 were then determined. The ratio of NCDH/NCD was significantly increased upon the addition of phosphite, indicating that more NCD existed in the form of NCDH. The ratio reached a maximum value of 2.05 at 20 min, which was 7.6-fold higher than that without phosphite supplementation. In contrast, upon phosphite supplementation, the alterations of NADH/NAD and NADPH/NADP ratios observed in strain XJD2701 were relatively modest (Figure 2b–d). Similar changing trends of NADH/NAD and NADPH/NADP ratios were also observed in the control strain XJD2801 incapable of NCD biosynthesis (Figure S4). These results indicated that Pdh* was able to selectively reduce NCD to NCDH and keep most NCD in the reduced form in vivo, while NAD and NADP were not obviously perturbed. However, it is worth noting that upon phosphite addition in strain XJD2701, NCDH constituted only around two-thirds of the cellular NCD pool at maximum, and a considerable amount of NCD remained in the oxidized form. We tried to increase the concentration of phosphite to 10 mM to reduce more NCD, but further reduction in NCD was compromised. This could be attributed to the relatively low transmembrane transport efficiency of phosphite, which prevented the complete reduction in NCD.

### 3.3. Reduction of NCD Driven by Phosphite in Cell-Free Systems

**Figure 3:**
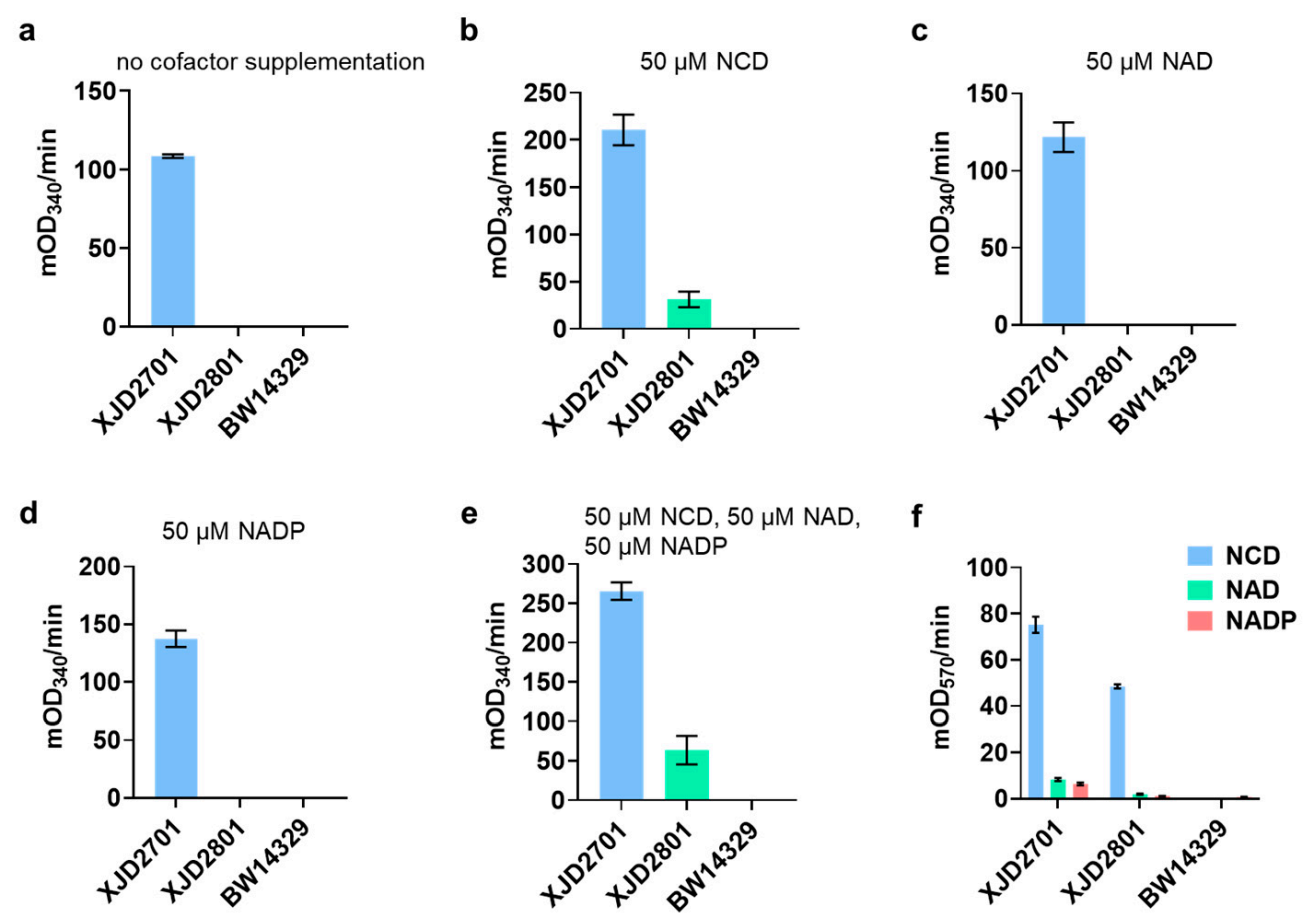
Cofactor reduction in cell-free systems and crude enzyme activity of Pdh* in engineered strains. (**a**–**e**) Crude enzyme activity of cell lysate. (**f**) Crude enzymatic activity of Pdh* towards NCD, NAD, and NADP. All measurements were conducted in triplicate.

To verify whether phosphite could drive the complete reduction in NCD, assays were carried out at the crude enzyme level. In the presence of phosphite, an increase in absorbance at 340 nm (OD_340nm_) was exclusively observed in the crude enzyme of XJD2701, indicating specific NCDH reduction (Figure 3a). Subsequently, 50 μM NCD, NAD, NADP, and a mixture of them were added to the cell lysate of XJD2701 alongside phosphite, respectively (Figure 3b–e). It showed that the addition of NCD resulted in a doubled reduction rate (Figure 3b). In contrast, supplementation of NAD or NADP yielded only a marginal increase, which was attributed to the residual activity of Pdh* towards these native cofactors (Figure 3f). These results suggested that the dominant reductive activity measured in the cell lysate of strain XJD2701 was attributed to the reduction in NCD. In addition, the mixed cofactor assay exhibited a slightly higher reduction rate (Figure 3b,e), showing a collective activity towards all three cofactors. For XJD2801, the reduction in NCD was significantly slower than that of XJD2701, and negligible NAD- or NADP-dependent activity was detected, which was consistent with the enzymatic activity assay.

To quantify the NCD reduction rate in cell lysate, the molar extinction coefficient of NCDH (ε_NCDH_) in the system was calculated. A standard curve was established using varying concentrations of NCD in the lysate of XJD2701, yielding an ε_NCDH_ of 3.98 mM^−1^ cm^−1^ based on the Lambert–Beer law (Figure S5). Consequently, NCDH concentration in the system with cell lysate was calculated as 37.86 μM (Figure 3a), and NCDH concentration in the system with the supplementation of 50 μM NCD was identified as 90.05 μM (Figure 3b). The concentration difference in NCD was 52.10 μM, which agreed well with the supplemented concentration of NCD. This confirmed the reliability of the detection system.

Given that the intracellular amount of NCD in XJD2701 was 749.5 μM (Figure S6), there was nearly 33.71 μM NCD in the reaction system. According to the calculated NCD concentration of 37.86 μM, it can be concluded that nearly all NCD was reduced in the presence of 5 mM phosphite in the cell-free system. These results further confirm that the incomplete reduction in NCD in vivo is primarily due to limited phosphite uptake, rather than insufficient Pdh* activity. This suggested that improving phosphite transport represented a key direction for enhancing the efficiency of NCD-mediated reductive processes in living cells.

### 3.4. Enhancement of Intracellular NCD Reduction via Cell Permeabilization Induced by Polymyxin B

**Figure 4:**
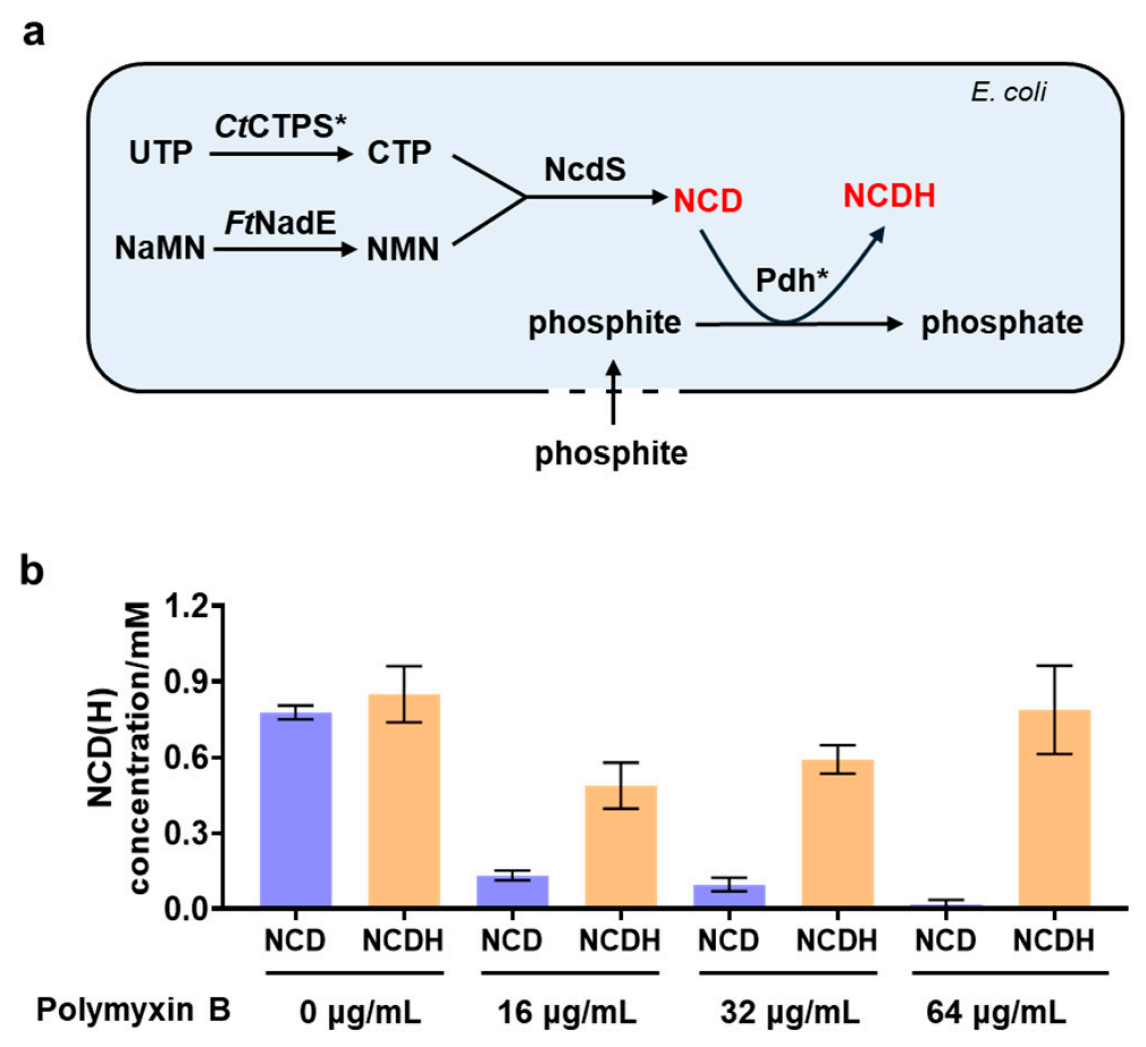
Enhancement of intracellular NCD reduction by polymyxin B-induced cell permeabilization. (**a**) Reduction in NCD in cells. *Ct*CTPS*, CTP synthetase mutant (D149E) derived from *Chlamydia trachomatis*; *Ft*NadE, nicotinamide mononucleotide synthetase from *Francisella tularensis*. (**b**) Concentration of NCD and NCDH. Cells were suspended in the system with 40 mM MOPS (pH 7.2), 10 mM phosphite and varying concentrations of polymyxin B (0, 16, 32, and 64 μg/mL). Samples were treated at 30 °C for 30 min.

To enhance the uptake of phosphite, we subsequently permeabilized the engineered cells using polymyxin B (Figure 4a). Polymyxin B disrupts outer membrane structures by creating transient pores and changes the permeability of the membrane [21,29]. We speculated that polymyxin B could facilitate the transmembrane transport of phosphite and subsequently promote intracellular NCD reduction.

In the control without polymyxin B supplementation, reduced NCD (NCDH) accounted for 52% of the total NCD(H) in 30 min. The proportion of NCDH rose gradually with increasing polymyxin B concentrations, indicating that polymyxin B enhanced cellular permeability. When the concentration of polymyxin B was 64 μg/mL, NCDH accounted for 98% of total NCD(H), demonstrating that nearly the entire NCD(H) pool was present as the reduced form (Figure 4b). The NCDH/NCD ratio reached 47, showing that robust regulation of the NCDH/NCD ratio was achieved via phosphite oxidation. In contrast, in the absence of phosphite, intracellular NCD(H) remained predominantly in the oxidized form (NCD), regardless of polymyxin B treatment (Figure S7). These data confirm that the reduction in NCD is strictly phosphite-dependent, and that polymyxin B serves to enhance phosphite transport without directly affecting the NCD redox state. Among the tested polymyxin B concentrations, 64 μg/mL was identified as optimal for balancing phosphite uptake and cofactor retention. At this concentration, NCD was almost completely reduced to NCDH, and the total intracellular cofactor loss was comparable to that observed at 16 μg/mL, indicating that increasing polymyxin B concentration from 16 to 64 μg/mL did not further compromise membrane integrity.

Notably, total levels of NCD and NCDH declined with polymyxin B treatment, implying partial leakage of intracellular NCD(H) from cells due to increased membrane permeability, which was verified by quantifying NCD(H) in the supernatant (Figure S8). To avoid the loss of total intracellular NCD(H), other approaches may be considered to enhance phosphite uptake without disrupting cell structure, including the overexpression of phosphite transporters or adjustment of culture pH to favor phosphite transport [30,31]. Collectively, these results showed that intracellular NCD was almost completely reduced, and cells maintained a strongly reductive intracellular environment.

## 4. Conclusions

In this study, we utilized phosphite as an energy source and achieved selective intracellular reduction in NCD via the NCD-dependent Pdh*. We first validated the accuracy of coupled enzymatic colorimetric assays for cofactor quantification. The feasibility of selective upregulation of intracellular NCDH/NCD ratio driven by phosphite was then demonstrated, confirming that reduction in NCD could be realized without perturbing the cellular NADH/NAD ratio and NADPH/NADP ratio. To overcome the limitation of phosphite uptake and further elevate the NCDH/NCD ratio, cell-free system assays were conducted and verified that phosphite could drive nearly complete NCD reduction with minimal interference with NAD and NADP. Finally, intracellular permeabilization treatment by polymyxin B facilitated phosphite uptake into cells, and resulted in 98% conversion of total cellular NCD to NCDH with NCDH/NCD ratio reaching 47. This work demonstrates that NCD can function as an independent cofactor for selective redox regulation in NCD-synthesizing engineered strains. These findings provide theoretical support and a viable strategy for energy-selective biosynthesis relying on the artificial cofactor NCD.

## Data Availability

The data supporting the findings of this study are available within the article and its Supplementary Information Files.

## Supplementary Materials

The following supporting information can be downloaded at: https://www.mdpi.com/article/10.3390/biom16091255/s1. Figure S1: SDS-PAGE analysis of purified proteins. Figure S2: Standard curves of different cofactors; Figure S3: Crude enzyme activity of engineered strains; Figure S4: Changes in NADH/NAD ratio and NADPH/NADP ratio in strain XJD2801; Figure S5: Standard curve of NCD concentration versus absorbance; Figure S6: Cofactor concentration in strain XJD2701; Figure S7: NCD(H) concentration in the absence of phosphite; Figure S8: NCD(H) concentration in the supernatant of the systems treated by Polymyxin B; Table S1: Strains and plasmids used in this study.
