# Supplementary material for: Selective Redox Tuning Enables Potent Intracellular Reduction of Nicotinamide Cytosine Dinucleotide
Source: Biomolecules. 2026 Aug 29;16(9):1255. doi: 10.3390/biom16091255 (PMC13604149; doi:10.3390/biom16091255)
Supplement: Supplementary file 1 [file biomolecules-16-01255-s001.zip › biomolecules-4511710-supplementary.pdf]

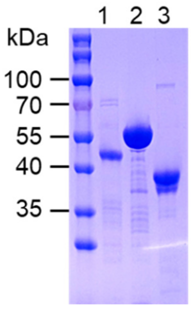

Figure S1. SDS-PAGE analysis of purified proteins. Lane 1, formate dehydrogenase mutant FDH*; lane 2, NADP-dependent glyceraldehyde-3-phosphate dehydrogenase (*Sm*GapN) from *Streptococcus mutans*; lane 3, fructose-bisphosphate aldolase class II (FbaA) from *E. coli*.

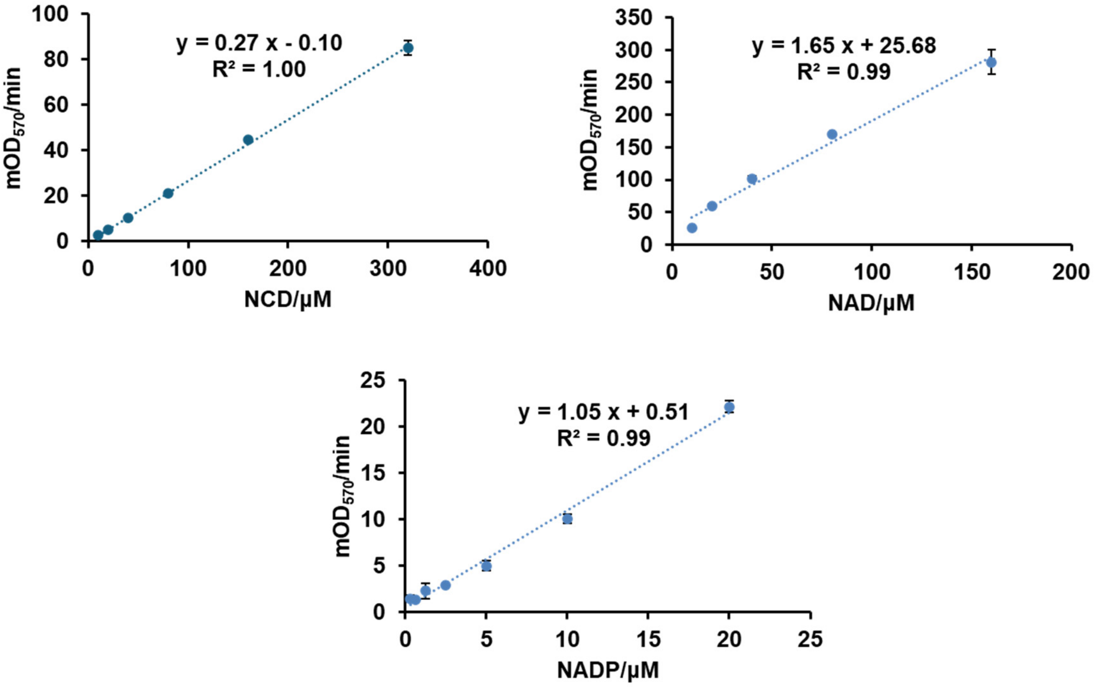

Figure S2. Standard curves of different cofactors. Based on the intracellular concentrations of NAD, NCD and NADP, the concentrations of NAD and NCD in the tested samples were estimated to be approximately 125 μM, while the NADP concentration was around 6 μM.

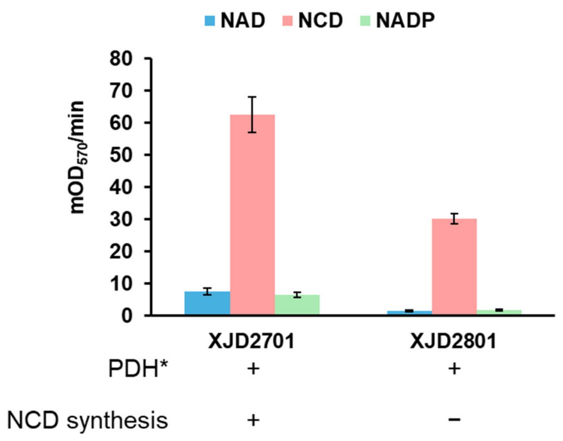

Figure S3. Crude enzyme activity of engineered strains.

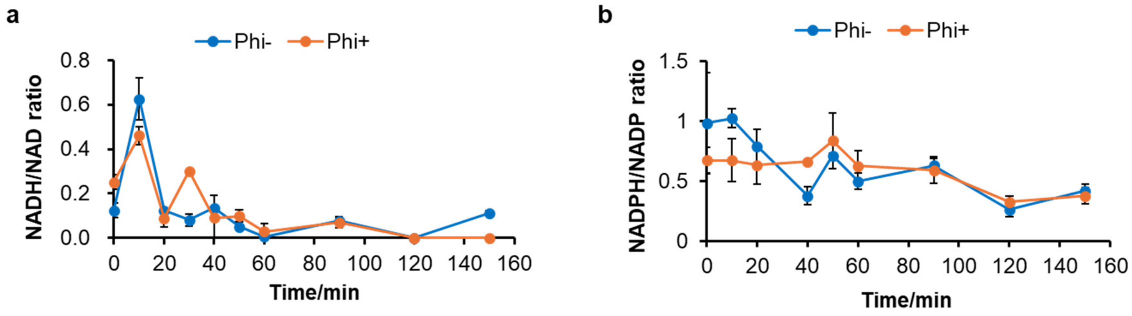

Figure S4. Changes of NADH/NAD ratio and NADPH/NADP ratio in strain XJD2801.

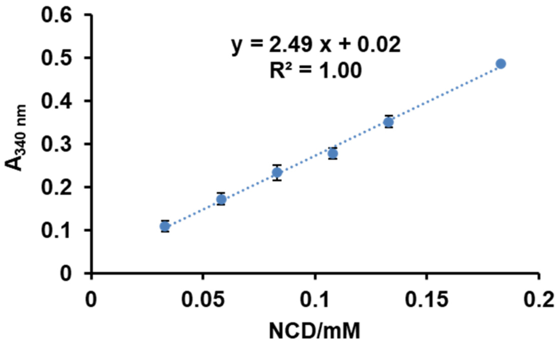

Figure S5. Standard curve of NCD concentration versus absorbance. The reaction system (total volume 200 μL) contained 50 mM HEPES (pH 7.5), 5 mM phosphite, and 180 μL cell lysate, with an optical path of 0.625 cm. Concentrations of NCD (mM) were set at 0.033, 0.058, 0.083, 0.108, 0.133 and 0.183.

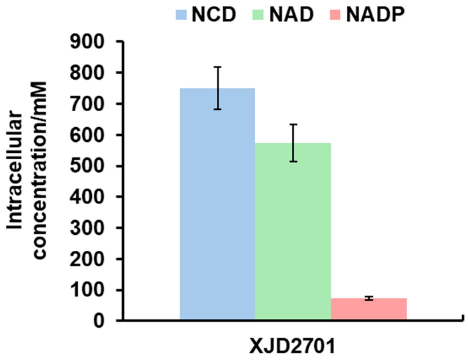

Figure S6. Cofactor concentration in strain XJD2701.

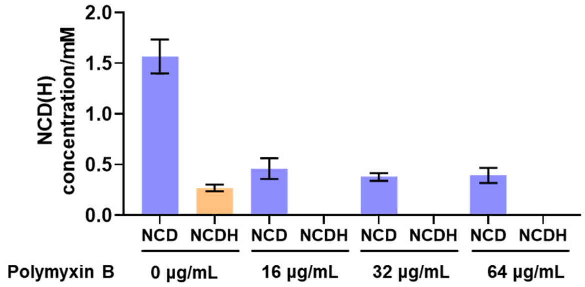

Figure S7. NCD(H) concentration in the absence of phosphite. Cells were suspended in the system with 40 mM MOPS (pH 7.2) and varying concentrations of polymyxin B (0, 16, 32, and 64 μg/mL). Samples were treated at 30 °C for 30 min.

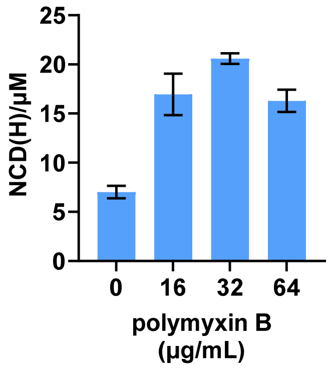

Figure S8. NCD(H) concentration in the supernatant of the systems treated by Polymyxin B.

Table S1 Strains used in this study.

Strains	Description	Source
BW14329	F$^-$, Δ*(argF-lac)169*, Δ*phoA532*, *λ*$^-$, *rpoS396*(Am), *rph-1*, Δ*(phnP-mel)522(Tn5-1/132)*, *hsdR514*, *creC510*, *Mu*$^+$	CGSC#8283
XJD2701	BW14329 harboring pk-2B3-1G6 and pUC-WXY63	This study
XJD2801	BW14329 harboring pk-2B3-1G6 and pUC-P15A	This study
Bl21(DE3) pET28a-*Sm*GAPN	Bl21(DE3) haboring pET28a with glyceraldehyde-3-phosphate dehydrogenase (*Sm*GAPN) from *Streptococcus mutans*	This study
